# 228. Increased Incidence of Invasive *Haemophilus influenzae* Infections in Adults in Central Massachusetts

**DOI:** 10.1093/ofid/ofad500.301

**Published:** 2023-11-27

**Authors:** Qadija H Qadri, Richard T Ellison, Doyle V Ward, Jungwon Yoon

**Affiliations:** University of Massachusetts Memorial Medical Center, Shrewsbury, Massachusetts; University of Massachusetts Chan Medical School, Worcester, Massachusetts; University of Massachusetts Chan Medical School, Worcester, Massachusetts; UMass Medical School, Worcester, Massachusetts

## Abstract

**Background:**

Since the advent of the *Haemophilus influenzae* B vaccine, the incidence of infections due to this pathogen has substantially decreased, although occasional cases of severe infections due to other serotypes are observed. Routine surveillance at an academic medical center in central Massachusetts identified an apparent increase in the incidence of community-acquired bacteremic *H. influenzae* infections in adult patients and further investigation was undertaken.

**Methods:**

A retrospective review was performed of all patients with positive blood or cerebrospinal fluid cultures for *H. influenzae* hospitalized at UMass Memorial Medical Center between January 1, 2008 and March 31, 2023. Available blood culture isolates of *H. influenzae* from recent bacteremic cases underwent whole genome sequencing (WGS).

**Results:**

There was a notable increase in the quarterly incidence of *H. influenzae* cases seen in the last quarter of 2022 (3 cases) and first quarter of 2023 (8 cases) as compared to the period from 2008 through the third quarter of 2022 (0.7 cases) [figure 1]. The 11 cases involved 2 children and 9 adults with the median age of adults 66 years. All cases were bacteremic with 8 associated with pneumonia and 1 with pharyngitis, with 2 other infections. Underlying conditions included emphysema, cancer, diabetes, Crohn's disease and others; 2 patients died. There was no apparent geographic clustering. Three isolates were available for WGS with two being in serotype F clade (HiF) and one a non-typeable clade. The two HiF isolates had 1210 SNV differences suggesting the same genomic clade, but not related by recent transmission.

**Figure:**
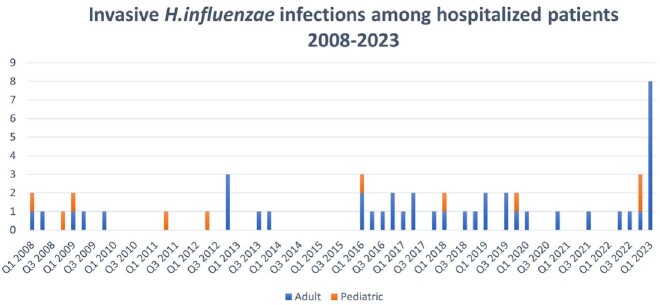

**Conclusion:**

A notable increase in invasive *H. influenzae* infections has been seen in our institution. The majority of the most recent cases have been in adults, and about 72% of the 11 cases were in association with pneumonia. Of the case fatalities, one was taking an IL-1 inhibitor, and another with cancer. Potential etiologies could be the emergence of a new and more invasive *H. influenzae* strain, as well as changes in either the host immunity to this pathogen or the upper respiratory tract microbiome in relation to the recent COVID pandemic and the effect of social isolation and increased use of face masks.

**Disclosures:**

**All Authors**: No reported disclosures

